# Discordance between CSF and PET biomarkers of amyloid pathology are associated with distinct pathophysiologic process in early stages of Alzheimer’s disease

**DOI:** 10.1002/alz.089461

**Published:** 2025-01-09

**Authors:** Binchong An, Danni Li, Lu Men

**Affiliations:** ^1^ University of Minnesota, Twin Cities, Minneapolis, MN USA; ^2^ University of Minnesota, Minneapolis, MN USA

## Abstract

**Background:**

Alzheimer’s disease (AD) pathophysiology is complex. Decrease in CSF soluble Aβ_42/40_ and accumulation of amyloid plaques (by amyloid PET) are two distinct biomarkers of amyloid pathology. However, the pathophysiologic process associated with each biomarker is not clear. To this end, we examined CSF protein difference across biological stages defined by biomarkers, with emphasize on early stages of AD where CSF and amyloid PET evaluations are discordant.

**Method:**

392 nondemented participants from the Alzheimer’s Disease Neuroimaging Initiative (ADNI) were biologically defined based on CSF Aβ_42_, amyloid PET, and CSF p‐tau into four groups (controls [Aβ_42_‐/ PET‐], Stage 1a [Aβ_42_+/ PET‐], Stage 1b [Aβ_42_‐/ PET+], Stage 2 [Aβ_42_+/ PET+]). Of note, Abnormal CSF Aβ_42_(+) was defined as < 976.6 pg/mL and normal (‐) as ≥ 976.6 pg/mL; abnormal brain amyloid by PET (+) was defined as ^18^F‐florbetapir > 1.11 SUVR and normal (‐) as ≤ 1.11 SUVR. All 4 groups had normal CSF p‐tau (defined as ≤ 26.47 ng/mL). SomaScan was used to measure over 7000 proteins in CSF proteome.

**Result:**

There was no significant protein difference between controls (Aβ_42_‐/PET‐) and Stage 1b (Aβ_42_‐/ PET+), or between Stage 1a (Aβ_42_+/ PET‐) and Stage 2 (Aβ_42_+/ PET+), after Benjamini‐Hochberg adjustment. We found 1462 proteins differentially expressed between controls (Aβ_42_‐/PET‐) and Stage 1a (Aβ_42_+/PET‐) related to abnormality in CSF soluble Aβ_42_. This number was much higher than 62 proteins differed between Stage 1b (Aβ_42_‐/ PET+) and Stage 1a (Aβ_42_+/PET‐) related to abnormality in amyloid PET. GO enrichment analysis of biological pathways and cellular components suggested neuropeptide signaling pathway and extracellular process are the most significant pathway and cellular components related to amyloid PET; whereas numerous pathways in neuron, synapse, axon were enriched and represented by protein differences related to CSF soluble Aβ_42._

**Conclusion:**

Our results reveal distinct pathophysiologic process associated with decrease in CSF soluble Aβ_42_ and increase in amyloid plaques measure by amyloid PET. Decrease in CSF Aβ_42_ appears to be more neurotoxic and are associated with more pathophysiological changes than increase in amyloid plaques at the early stages of AD.

